# Supplementary material for: How, and under what contexts, do academic–practice partnerships collaborate to implement healthcare improvement education into preregistration nursing curriculums: a realist review protocol
Source: BMJ Open. 2023 Oct 24;13(10):e077784. doi: 10.1136/bmjopen-2023-077784 (PMC10603429; doi:10.1136/bmjopen-2023-077784)
Supplement: Author's manuscript [file bmjopen-2023-077784.draft_revisions.pdf]

BMJ Open

BMJ Open is committed to open peer review. As part of this commitment we make the peer review history of every article we publish publicly available.

When an article is published we post the peer reviewers' comments and the authors' responses online. We also post the versions of the paper that were used during peer review. These are the versions that the peer review comments apply to.

The versions of the paper that follow are the versions that were submitted during the peer review process. They are not the versions of record or the final published versions. They should not be cited or distributed as the published version of this manuscript.

BMJ Open is an open access journal and the full, final, typeset and author-corrected version of record of the manuscript is available on our site with no access controls, subscription charges or pay-per-view fees (http://bmjopen.bmj.com).

If you have any questions on BMJ Open's open peer review process please email info.bmjopen@bmj.com

BMJ Open

# How, and under what contexts, do academic-practice partnerships collaborate to implement healthcare improvement education into preregistration nursing curriculums: a realist review protocol.

| | |
|---|---|
| Journal: | *BMJ Open* |
| Manuscript ID | bmjopen-2023-077784 |
| Article Type: | Protocol |
| Date Submitted by the Author: | 14-Jul-2023 |
| Complete List of Authors: | Armstrong , Lorraine ; University of Stirling, Sport and Health Sciences<br>Moir, Chris; University of Otago, Centre for Post Graduate Nursing Studies<br>Taylor, Peta; Ara Institute of Canterbury Ltd , Department of Health Practice |
| Keywords: | Health Education, Systematic Review, Nursing Care, Quality in health care < HEALTH SERVICES ADMINISTRATION & MANAGEMENT |
| | |

SCHOLARONE™
Manuscripts

**Title**: How, and under what contexts, do academic-practice partnerships collaborate to implement healthcare improvement education into preregistration nursing curriculums: a realist review protocol.

Corresponding Author:

**Mrs Lorraine Armstrong RN (A), (BSc).**

Lecturer in Nursing

School of Health Sciences

University of Stirling

Scotland

FK9 4LA

Tel: 01786 466117

Email: lorraine.armstrong@stir.ac.uk

ORCHID 0000-0001-8951-2712

**Dr Chris Moir RN, (PhD).**

Clinical co-ordinator/Senior Lecturer

Centre for Post Graduate Nursing Studies

University of Otago

8140 Christchurch

New Zealand

Tel: +64 3 364 3869

Email: chris.moir@otago.ac.nz

ORCHID 0000-0002-9178-2540

**Dr Peta Taylor RN, (PhD).**

Senior Nursing Lecturer

For peer review only

Department of Health Practice
Ara Institute of Canterbury Ltd
8140 Christchurch
New Zealand
Tel: +64 3 940 7556
Email: peta.taylor@ara.ac.nz
ORCID 0000-0002-9182-8254

For peer review only

## Abstract

**Introduction**: Internationally, healthcare improvement remains a clinical and educational priority. Consensus in Europe, Canada and the USA to implement quality improvement (QI) education into preregistration nursing curricula ensures students become equipped with the skills and knowledge required to improve practice. Now, New Zealand and Australia are beginning to implement QI education into their nursing curricula. However, QI education is complex; comprising multiple components, each influenced by the contexts under which they are developed and implemented. Evaluation studies of QI education unanimously acknowledge that academic and practice partnerships (APPs) are essential to optimally embed QI into preregistration curricula, yet it is not understood how, and under what contexts, APPs collaborate to achieve this.

**Methods and analysis**: A realist review to determine how, and under what contexts, APPs collaborate to implement QI education in pre-registration nursing will be conducted using the RAMSES Guidelines. International stakeholders will be consulted at each stage which includes 1) clarifying the scope of the review through empirical literature and tacit expert knowledge, 2) searching for evidence in healthcare and social science databases/grey literature, 3) appraising studies using the EPPI weight of evidence framework and extracting data using SQUIRE-EDU Publication Guidelines, 4) synthesising evidence and drawing



For peer review only - http://bmjopen.bmj.com/site/about/guidelines.xhtml

conclusions through the creation of context, mechanism and outcome configurations and 5) disseminating findings through conferences and peer-reviewed publications.

**Ethics and Dissemination**: Ethical approval was not required for this study. Findings will be disseminated to international nurse educators, leaders and front-line staff implementing QI education within their own academic and practice contexts through conferenced and peer-reviewed publications.

**Prospero Registration Number**

CRD42021282424

**Keywords**

Realist Review, Pre-registration Nursing Education, Academic-practice Partnerships, Context, Quality Improvement

**Strengths and limitations of this study**

- Realist review is novel to explore QI education in preregistration nursing.
- Understanding academic-practice partnerships in developing QI education is novel.
- Focus on the influence of context on QI education builds on the current literature.
- A limitation of the study is that only English articles will be included.

## Introduction

Healthcare improvement in nurse education

This millennium has witnessed a surge in healthcare improvement practice and educational development in response to practice errors and increased risk to patients' safety (Institute of Medicine, 2001). Therefore, developing workforce capacity and capability in improvement knowledge, skills and behaviours remains a priority on policy and educational agendas internationally (Scottish Government, 2010; Health Foundation, 2012; NMC, 2019, Academy



For peer review only - http://bmjopen.bmj.com/site/about/guidelines.xhtml

of Medical Royal Colleges, 2016, QSEN 2010). In the past decade, countries such as the United Kingdom (UK), Sweden, Spain, Canada and USA have developed consensus that QI education in preregistration nursing curricula should be a regular and ongoing process, if students are to contribute to and lead practice improvements (NMC 2019, Smith et al. 2019, Lillo-Crespo et al. 2017, Flynn et al. 2017, QSEN 2010). Recently, New Zealand has begun to embark on QI curricular integration, having established from 49 educational institutions that few display the foundations of quality and safety education (Robb et al. 2017). It is recognised that failure to address this gap compromises graduate nurses' abilities to implement and sustain healthcare improvement. A similar drive for QI education in pre-registration nursing curricula has also emerged from Australia where much of the focus is around partnership working (Mak et al. 2022). The authors of this review protocol have integrated, or are about to integrate, QI curricula into their nurse education programmes in the UK and New Zealand and have a shared purpose to develop valuable insights into how APPs collaborate to optimally embed QI into nurse education.

To date QI educational content in preregistration nursing has included improvement methods, quality indicator measures, the Model for Improvement, Plan-Do-Study-Act cycles, root cause analysis, systems thinking, inter-professional learning, clinical governance, data, human factors and evidence-based practice, which, when delivered through an experiential learning pedagogical approach, is regarded most appropriate to equip pre-registration students with the necessary skills and knowledge about QI. Supplementary approaches include didactic learning, seminars, group work, self-directed learning and/or simulation (Armstrong et al. 2017).

QI education is regarded as a complex social phenomenon, whereby its numerous components such as the learner, the curricula, the faculty and the practice setting all interact on multiple levels of the system. They are also subject to the varying contexts under which they are developed and implemented (Jones et al. 2014). Considering QI education from a *'systems thinking'* perspective is useful here, because this approach views the components as a connected whole rather than separate component parts (Health Foundation 2010).



For peer review only - http://bmjopen.bmj.com/site/about/guidelines.xhtml

There are many component parts which contribute to the complex nature of developing and implementing preregistration QI nurse education. The literature describes these as: developing the curricula, delivering teaching content, creating real life practice-based QI opportunities, developing staff expertise in improvement methodologies, securing mentorship and support, bridging support through information provision and link staff, updating student mentors on QI education through workshops, developing resource handbooks, seeking approval of students' improvement ideas, access to data, governance of ethical student practice, creating a QI culture where practice staff facilitate and mediate between students and other staff members, ongoing development and evaluation of QI education, and lastly supporting dissemination activities to showcase students QI work (Baillie et al. 2014, Christiansen et al. 2010, James et al. 2014, Starr et al. 2016). Taking account of these multiple components, it is evident that developing and implementing complex QI educational curricula requires synchronised planning between academics and practitioners, if students are to engage in meaningful practice-based improvement opportunities.

Despite a growing interest emerging from academics and practitioners to implement QI nurse education, there is currently no research to inform how, and under what contexts, APPs collaborate to achieve this (Robb et al. 2017, Beaver 2019, Mak et al. 2022).

<u>Academic-practice partnerships</u>

Academic-practice partnerships (APPs) are defined as '*strategic associations between educational and clinical facilities that are established to advance their mutual interests in nursing practice, education and research'* (Beal 2012). Their purpose ranges from developing preceptorship education to clinical nurse leadership initiatives in hospitals, community health and public health agencies, nursing homes, schools, and governmental agencies (Macphee 2009, Beal 2012). They operate on strategic, tactical, operational, interpersonal and cultural levels (Brown 2006).



For peer review only - http://bmjopen.bmj.com/site/about/guidelines.xhtml

There has been a recent review that considered the effectiveness of nurse APPs on costs, employability, work-readiness, confidence, competence and stakeholder satisfaction, but not in developing or implementing nurse education (Nguyen 2020). The same review also encourages future research to focus on *how* APPs function and sustain under different contexts. As such, how APPs function and sustain under different contexts when developing and implementing QI nurse education remains our focus.

We do know though that nursing APPs have been investigated in the management literature which attribute several key principles to successful collaboration (Brown 2006). These include creating a shared vision, establishing accountability, nurturing appropriate people engagement with balanced relationships, maintaining trust and respect, practising good communication and putting governance in place. Through these principles, APPs benefit from improved organisational efficiencies, enhanced opportunities for innovation and enhanced recruitment and retention (Brown 2006).

Similarly, there is evidence from the management literature of investigations into how different types of partners in healthcare improvement practice and research, not education, generally work together (Kaplan et al. 2010). It is suggested that healthcare improvement partners require three types of skills to function and be sustained. These relate to technical skills (Lean, PDSA, run charts), soft skills (communication, leadership, team skills) and learning skills (knowledge sharing, encouraging participation and collective learning). In the absence of one skill subset, it is reported that healthcare improvement partners experience difficulties leading change (Health Foundation, 2014).

Lastly, a scoping review collating evidence of the effectiveness of partnerships in clinical nurse education has also been undertaken; however, the eligible studies in this review adopt weak evaluation measures and use low-quality appraisal instruments to determine overall effectiveness (Nguyen 2020).

If nursing APPs are to develop and implement QI education under their own practice and education contexts, then insights into the experiences of this process is essential.



For peer review only - http://bmjopen.bmj.com/site/about/guidelines.xhtml

What will a realist review add to the field?

Realist methodology is a theory-driven approach appropriate for investigating how APPs work together, because it not only considers the complex nature of QI education which operates as a social intervention but recognises the different contexts and mechanisms that contribute (or not) to the QI educational programme's success (Pawson et al. 2004). The Medical Research Council (MRC) have recently updated their complex intervention guidelines to encourage realist approaches for evaluation studies (Skivington et al. 2021).

A previous review that has investigated the effectiveness of APPs working in clinical education, and one examining the methodological rigour in QI curricula, both criticise studies for the research approaches they have adopted (Nguyen 2020, Windish et al. 2009). This is because, predominantly, researchers investigating QI education have asserted a positivist stance. Traditionally, positivists sit within the natural (physical) sciences, are associated with quantitative approaches and claim that an absolute truth of knowledge exists (Denzin and Lincoln 2005).

Realist researchers also sit within the natural (physical) sciences as post-positivists but challenge an absolute truth theory in favour of a realist perspective. Both sides proclaim broadly similar *effect* and *outcome* orientations, but post-positivists argue that only an approximation of truth exists, and as such focus on the mechanistic factors which determine outcomes, as opposed to the outcomes themselves (Creswell 2014).

Positivist stances and quantitative approaches are regarded as too simple to determine causal linkages between complex interventions and outcomes because they discount the all-important concept of context (Bates, 2014). Ignoring context is considered to be the antithesis of healthcare improvement principles and is instead a pre-requisite to understanding why success ensues in one instance and not in another. The context debate is infinitely evolving, and in-depth discussions are widely available (Health Foundation 2014, Kaplan et al. 2010). However, in general terms it relates to *everything*. Context has become the central focus of QI nurse education studies recently, in which ethnographic methodology



For peer review only - http://bmjopen.bmj.com/site/about/guidelines.xhtml

is used to explore the contexts that influence student nurses QI learning experiences in the practice setting (Armstrong et al. 2019).

There have been many frameworks developed to study context in healthcare improvement (Coles et al. 2020). For instance, The Model for Understanding Success in Quality improvement (MUSIQ) targets contexts relating to micro-systems; these include the QI team, QI support and capacity, the organisation, and the external environment (Kaplan et al. 2012) while others consider context as the *outer* components (political, social and cultural) and *inner* components (organisational structure, size and performance) (Bate 2014). In terms of realist reviews, Greenhalgh and Manzano (2021) have more recently identified context as 1) tangible, fixed, observable features that trigger mechanisms or 2) relational and dynamic features that shape the mechanisms through which the intervention works.

For the purpose of exploring *'contexts'* this review protocol will draw upon Tess et al.'s (2015) conceptual framework of integrating quality and safety into medical education. We did not come across a similar model which exists for nurse education in the literature. The concepts in their model will form our preliminary investigation into the contexts APPs might operate under. The contexts we are initially guided towards, but will not be restricted by, are organisational culture, academic-practice alignment, infrastructure, curricular resources, faculty-practice development and inter-professional collaboration (see Table 1). As our review progresses and our data evolves, we will develop context themes iteratively and report upon any further developments in the final write up of the study.

- Insert Table 1 here

## Methods and analysis

For completeness and transparency this review protocol has upheld the recommendations of PRISMA-P (Preferred reporting items for systematic review and meta-analysis protocols),



For peer review only - http://bmjopen.bmj.com/site/about/guidelines.xhtml

where it relates to our realist review design and is available in additional file 1 (Moher et al. 2009).

Study Design

Our realist review of the literature will be guided and conducted in line with the Realist and Meta-narrative Evidence Syntheses: Evolving Standards (RAMSES) Guidelines (Wong et al. 2013). This approach will enable a rigorous process to be followed ensuring that both methodological decisions remain appropriate to the review type and anticipated data set. The steps are 1) clarifying the scope of the review through empirical literature and the authors' tacit expert knowledge, 2) searching for evidence in healthcare and social science databases, and grey literature, 3) appraising studies using the Evidence for Policy and Practice Information and Co-ordinating Centre (EPPI) weight of evidence framework and extract data using SQUIRE-EDU (Standards for QUality Improvement Reporting Excellence in Education): Publication Guidelines, 4) synthesising evidence and drawing conclusions through the creation of context, mechanism and outcome configurations and 5) disseminating findings through conference presentations and peer-reviewed publications.

Study Methods

**Clarifying the scope**

The original scope of our realist research stems from the findings of our previous research. Firstly, an integrative review which explored QI education in pre-registration healthcare examined QI educational interventions in pre-registration healthcare. This study concluded that there was a need to develop and strengthen APPs to embed QI in nurse education (Armstrong et al. 2017). Equally, a drive from New Zealand and Australian healthcare sectors to embed QI into their nursing programmes has broadened the scope of the review and created an opportunistic and timely collaboration to develop insights into how APPs have implemented QI education internationally (Robb et al. 2017, Beaver 2019, Mak et al. 2022). This evolving landscape, coupled by the authors' academic and clinical tacit



For peer review only - http://bmjopen.bmj.com/site/about/guidelines.xhtml

knowledge of developing and implementing QI nurse education, has shaped the initial aim and objectives early in the process.

*Study Aim*

The aim of our realist review is to develop an understanding of *'how'* and under what contexts APPs collaborate to implement healthcare improvement education into pre-registration nursing curriculums.

*Study Objectives:*

The key objectives of the research review are to:

1. Identify the contexts under which APPs collaborate to implement QI into preregistration nurse education
2. Analyse the mechanisms and outcomes associated with each context under which APPs collaborate to implement QI into preregistration nurse education
3. Explore relationships emerging across and between the contexts, mechanisms and outcomes in which APPs collaborate to implement QI into preregistration nurse education
4. Explain the causal pathways of how APPs collaborate to implement QI into preregistration nurse education
5. Disseminate findings so that nursing and academic institutions, new to QI education, are encouraged and well informed to develop their own highly effective APPs

The next step entailed undertaking a broader exploration of the literature to enable us to refine the review's parameters. We identified a key difference between international countries; that being some healthcare systems such as the UK, New Zealand, Australia and Canada, were publicly funded compared to that of the USA which is privately funded. Exploring both healthcare systems was believed to be too vast for the team's time and resource capacity. However, our scoping activity retrieved lower-than-expected numbers of studies from outside of the USA (who remain international leaders in healthcare



For peer review only - http://bmjopen.bmj.com/site/about/guidelines.xhtml

improvement education and research). As a result, we broadened the review's international range and will contemplate whether this difference in healthcare systems could act as a potential '*context'* category under which APPs collaborate.

Lastly, the core research team held a stakeholder engagement exercise to assist in the development of the study in December 2021. Stakeholders that were contacted included international academic and practice staff operating on multiple levels of the healthcare and academic organisations such as pre and post registration nurses, nurse directors, nurse academics, clinical nurse educators, nursing directors of quality and nursing journal editors. We provided an opportunity to shape the realist review protocol, make adaptations to the study methods, discuss pressing issues, and then actioned suggestions to refine the scope of the review. Stakeholders are listed in the acknowledgements. Consideration of the value that patients, carers and the public add to the project will be regularly reviewed by the team, acted upon for input where applicable and reported in the final review manuscript (Abrams et al. 2020).

**Developing our programme theory**

Programme theories in realist review relate to the intervention under investigation, in this case the QI educational programme. They seek to offer a theoretical explanation of how the intervention works, for whom, and under what contexts. Seeking out assumptions of how interventions operate under different contexts, through a variety of mechanisms which produce different outcomes, is a continuous, collaborative and an iterative process which uses theoretical and empirical literature to populate and test initial '*rough theories'* (Wong et al. 2013). A number of initial rough theories about how APPs collaborate to implement healthcare improvement education have influenced our investigation:

- Experiential learning is the most appropriate pedagogical approach to equip student nurses with adequate QI knowledge and skills. Therefore, we



For peer review only - http://bmjopen.bmj.com/site/about/guidelines.xhtml

anticipate that early formation of APPs is needed to streamline quality teaching and learning experiences.

- The international landscape varies, and some APPs are more experienced at implementing QI education than others. It is possible that policy drivers and/or professional standards from within each country will determine to a greater/lesser extent, the priority by which QI education is implemented.

- APPs operate on multiple levels of the organisation just as QI education comprises multi components that requires a '*systems thinking*' perspective. We predict that multiple APPs may operate simultaneously, over multiple levels of the organisation to implement one QI education programme, rather than one APP operating on an individual basis.

In line with realist review methods, our '*initial rough theories*' stated above, will continue to be refined and/or expanded in an iterative manner as we explore further literatures, and analyse and populate our data into our context framework (Wong et al. 2013).

**Search for evidence**

To identify empirical studies relevant to the review question, a formal search of a range of databases in the social sciences and healthcare were undertaken.

*Electronic databases*

Electronic databases: Cumulative Index to Nursing and Allied Health Literature (CINAHL), EMcare, Medical Literature Analysis and Retrieval System Online (MEDLINE), Science Direct and Scopus were systematically searched. Study limitations included only peer-reviewed and English language studies. Aligning to the inception of QI nurse education in the millennium, databases were searched from 2000-2021. A primary search for MEDLINE



For peer review only - http://bmjopen.bmj.com/site/about/guidelines.xhtml

was developed using MeSH (Medical Subject Headings) terms. Subsequent searches were translated to each database through selection of controlled terms and Boolean terms, truncation, wildcards and proximity searching to maximise relevant papers. Search terms comprised variations of keywords relating to healthcare improvement, curriculum, education, pre-registration nursing, academic-practice partnership, context and implementation. A primary sample search strategy from MEDLINE is presented (see Table 2).

*Reference list searching*

The reference lists of eligible studies were fully scanned to identify any potentially relevant studies not detected initially by the search strategy. Where difficulties in retrieving studies were encountered, authors were contacted to request a copy of the article.

**Inclusion/Exclusion Criteria**

*Inclusion*

We included all international peer-reviewed studies of all designs that had an available abstract written in the English language from 2000 through to 2021. Studies were included if they described any element of APPs collaboration to implement QI education in preregistration nursing. We accepted and included '*quality improvement education*' as one that described the teaching, learning or utility of a healthcare improvement model by preregistration nursing students which detailed both a theory and practice-based element.

*Exclusion*

We excluded studies which made no reference to how the APPs collaborated, where they referred solely to online learning, patient safety content or did not include preregistration nursing students.

- Insert Table 2 here

**Data Collection and Management**



For peer review only - http://bmjopen.bmj.com/site/about/guidelines.xhtml

*Search for evidence*

Reviewers (LA, CM and PT) conducted and saved searches within corresponding databases. To ensure each search remains accessible, reproducible and transparent a manual record keeping log was produced. To allow reviewers access, studies eligible for screening were uploaded electronically to a shared named folder within Microsoft Teams. Duplicates were removed.

*Screening and selection of studies*

Reviewers (LA, CM and PT) worked independently to screen titles and abstracts from retrieved studies using predetermined inclusion and exclusion criteria. Ambiguities around papers were discussed and the decision to include or exclude them was made jointly. Articles for inclusion were transferred to a separate shared named folder to allow reviewer access for data extraction. Some excluded studies remained valuable to develop our initial theory of APPs, and for later discussion of the review findings. These articles were stored in a separate share folder in Microsoft Teams. To ensure consistency in the application of inclusion and exclusion criteria, one reviewer with subject expertise (LA) reviewed a 10% random sample of the same papers to confirm inter-rater reliability. Any discrepancies were resolved by discussion. The iterative nature of realist review searching means that papers may continue to be included through various means, therefore the final results yielded from each phase of the search strategy will be illustrated in a flow diagram once saturation is deemed to be reached and a final search is undertaken (Pawson et al. 2005).

*Data extraction & appraising the evidence*

Reviewers (LA, CM and PT) will extract data with cognisance of SQUIRE-EDU (Standards for QUality Improvement Reporting Excellence in Education): Publication Guidelines for Educational Improvement (Ogrinc et al. 2019). Reviewers will also focus on extracting data under the headings which relate to the key *'contexts'* of interest from Tess et al.'s (2015) framework. These include the organisational culture, practice-academic alignment, infrastructure, curricular resources, faculty-practice development and inter-professional collaboration. Mechanisms relating to each *'context'* will be extracted and included under

each heading. Reviewers will also extract the outcomes of interest that are detailed by authors in each study. One reviewer with expert subject knowledge (LA) will compare a 10% random sample to ensure consistency. Discrepancies will be resolved by discussion or a fourth reviewer where necessary.

A realist review is predicted to retrieve heterogeneity in study design, therefore using a singular hierarchical appraisal tool for exclusion will not be fit for purpose or add value to answering the study's questions. The EPPI Weight of Evidence framework which considers the use of judgement in relevance and quality will be used (Table 3). This framework allocates each study with a weight of high, medium or low in relation to three key areas: a) Trustworthiness of results of study, b) appropriateness of study design to the review question and c) appropriateness of focus to answering the review question. The results of a), b) and c) are combined and given an overall weight. This method allows the worth of each study to be identified for the realist synthesis rather than being a preliminary pre-qualification exercise (Gough 2007). To ensure consistency in overall Weight of Evidence allocated, reviewers will meet to compare a 10% random sample of low, medium and high graded papers and discrepancies resolved through discussion or a fourth reviewer if necessary.

- Insert Table 3 here

*Analysis and synthesis of evidence*

As a general framework, the Realist and Meta-narrative Evidence Syntheses: Evolving Standards (RAMSES) Guidelines will be applied (Wong et al. 2013). Analysis in realist reviews is primarily concerned with refining theory-driven explanations, in this case, of how APPs work collaboratively to implement QI education under different contexts. Analysis will involve, firstly: identifying contexts under which APPs collaborate and informed but not limited by a preliminary framework of QI education developed by Tess et al. (2015).  We will discuss new emerging contexts and consider what supporting evidence warrants its



For peer review only - http://bmjopen.bmj.com/site/about/guidelines.xhtml

inclusion to refine our programme theories. In line with *systems thinking,* we will explore the relationships that exist as a collective whole, in order to create a broader perspective of QI education as a holistic system and add depth to our understandings.

Next, the mechanisms and outcomes associated with each context will be investigated and listed as context, mechanism and outcome (CMO) configurations. A CMO configuration '*is a statement, diagram or drawing that spells out the relationship between particular features of context, particular mechanisms and particular outcomes'* (Wong et al. 2013 p13). The causal relationships within each CMO configuration will be explored and listed. Recurring themes will be identified and grouped as demi-regularities (semi-predictable patterns). We will consider how the evidence supports or disproves our initial rough theories of how APPs collaborate to implement QI education into preregistration nurse education. The findings will be presented as a narrative synthesis which the contexts and any relationships between them will be discussed. The demi-regularities associated with each context will be embedded into this discussion as we see appropriate, and our conclusions will take account of the current literature landscape to make future recommendations.

*Disseminate, implement and evaluate*

The research will be disseminated through published works, local and international conference presentations. The findings will be used by two New Zealand tertiary institutions and their partnering district health boards to develop effective APPs to implement QI education in their nursing programmes. A realist process evaluation will be undertaken to advance the field of QI education and continue knowledge development around APPs working.

## Ethics and dissemination

No ethical approval was required to undertake this study. This realist review is the first to our knowledge that aims to develop an understanding around how, and under what contexts, APPs collaborate to implement QI education in pre-registration nursing curriculums. This



For peer review only - http://bmjopen.bmj.com/site/about/guidelines.xhtml

study is aimed at international nurse educators and nurse leaders who can make sense of the findings to adapt their implementation approach within the limits of their own education and practice contexts. We will disseminate findings through conference presentations and peer-reviewed journals.

*Author Statement*

LA, CM and PT made substantial contributions to the conception and design of the protocol development. All authors have approved the submitted version and have agreed to be personally accountable for own contributions and ensure questions related to the accuracy or integrity of the work, even ones in which the author was not personally involved, are appropriately investigated, resolved, and the resolution documented in the literature.

*Funding statement*

This research received no specific grant funding from any funding agency in the public, commercial or not-for-profit sectors.

*Competing interests*

The authors declare that they have no competing interests.

*Word count*

3836

*Acknowledgements*

We would like to acknowledge the following stakeholders who contributed to the development of this realist review protocol.



For peer review only - http://bmjopen.bmj.com/site/about/guidelines.xhtml

- Jen Gow (RN) Nurse Consultant for Quality and Performance Improvement: Southern District Health Board, New Zealand
- Verity Mak (Med, MN, BN, Lecturer): Monash University, Victoria, Australia
- Professor Maura McPhee (RN, PhD, FCNEI): University of British Columbia, Canada
- Henry Ritche - (RN, MNSc): Wellington Regional Hospital, New Zealand
- Angela Williams (Mrs.) Senior Lecturer in Nursing (Adult): Wrexham Glyndwr University, Wales, United Kingdom
- Susan Wood (RGON BSc AdvN MNS FCNA, Director of Quality and Patient Safety): Te Whatu Ora Waitaha Canterbury and Te Tai o Poutini West Coast New Zealand

*Data Statement*

The datasets used and/or analysed during the current study are available from the corresponding author on reasonable request.

Table 1. Tess et al.'s (2015) framework for integrating QI education.

For peer review only



For peer review only - http://bmjopen.bmj.com/site/about/guidelines.xhtml

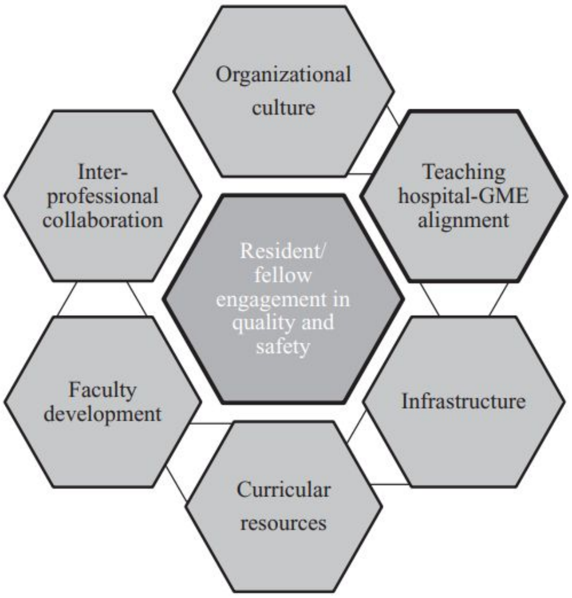


**Figure 1** Overview of a framework for integrating the quality and safety mission of a teaching hospital and graduate medical education (GME). The framework's six key elements are organizational culture, teaching hospital–GME alignment, infrastructure, curricular resources, faculty development, and interprofessional collaboration.

Tess et al. (2015)

Table 2. Developed Search Strategy for MEDLINE using OVID

1. Quality improvement/



For peer review only - http://bmjopen.bmj.com/site/about/guidelines.xhtml

2. Quality improvement.tw.
3. Quality Assurance, Health care/
4. Quality assurance.tw.
5. improvement science.tw.
6. improvement methodology*.tw.
7. Improvement model*.tw.
8. service improvement.tw.
9. CQI.tw.
10. plan do study act.tw.
11. PDSA.tw.
12. Plan do check act.tw.
13. PDCA.tw.
14. Total Quality Management/
15. Total quality management.tw.
16. TQM.tw.
17. 1 or 2 or 3 or 4 or 5 or 6 or 7 or 8 or 9 or 10 or 11 or 12 or 13 or 1 or 14 or 15 or 16
18. Interinstitutional Relations/
19. Interinstitutional relations*.tw.
20. Public-Private Sector Partnerships/
21. Public-Private partner*.tw.
22. academic practice partner*.tw.
23. intersectoral collaboration?.mp (mp=title, abstract, original title, name of substance word, subject, heading word, floating sub-heading word, keyword heading word, organism supplementary concept word, protocol supplementary concept word, rate disease supplementary concept word, unique identifier, synonyms)
24. collaborat*.tw.
25. communit* of practice.tw.
26. 18 or 19 or 20 or 21 or 22 or 23 or 24 or 25

For peer review only



For peer review only - http://bmjopen.bmj.com/site/about/guidelines.xhtml

27. Health Personnel/ed (Education)

28. Health personnel education.tw.

29. Health staff education.tw.

30. Exp Education, Nursing/

31. Nursing education.tw.

32. allied health education.tw.

33. or 28 or 29 or 30 or 31 or 32

34. 17 and 26 and 33

35. Limit 34 to (English language and yr=”2000-Current”)

36. From 35 keep

1, 12, 14, 28, 37, 43, 57, 66, 71, 74, 81, 94-96, 102-103, 117, 123, 128, 130-131, 136, 140, 144, 147, 149, 151, 163, 169, 174, 198, 219

Table 3. EPPI Weight of Evidence

| | WoE A | WoE B | WoE C | Overall WoE |
|---|---|---|---|---|
| **Weight of Evidence** | **Trustworthiness of results of study** | **Appropriateness of study design to review question** | **Relevance of study focus to answer review question** | **A,B & C Combined** |
| | ***High:*** **1.Intervention clearly articulated (who, what when, where, why) to allow for reproducibility. 2.Ethical, explicit and detailed methods sections for recruitment, data collection & analysis.** | ***High:*** **Large-scale multi-method designs e.g., RCT's, quasi-experimental using validated tools and/or interview, observational data with large sample size.** | ***High:*** **Provides useful & relevant answers closely relatable to review objectives.** | ***High:*** **A must be accredited no less than a 'high' in two domains to achieve an overall WoE of High.** |



For peer review only - http://bmjopen.bmj.com/site/about/guidelines.xhtml

| | | | | |
|---|---|---|---|---|
| | 3.Comprehensive reporting of data and accurate interpretation clearly warranted from findings. | | | |
| | ***Medium:*** 1.Intervention described but not with sufficient detail to replicate. 2.Satisfactory reporting of methods sections for ethics, recruitment, data collection & analysis - minor omissions evident. 3.Minor bias in reporting of data and interpretations partially warranted from findings. | ***Medium:*** Medium-scale qualitative, quantitative or mixed method designs e.g., local pre-post, post survey/questionnaire using acceptable tools and/or interview, observational data with medium/small sample size. | ***Medium:*** Provides answers relatable to review objectives. | ***Medium:*** A must be accredited no less than a 'medium' in 2 domains to achieve an overall WoE of Medium. |
| | ***Low:*** 1.Intervention not described. 2.Unsatisfactory methods sections for data collection and analysis with major omissions evident. 3.Unsatisfactory reporting of data and/or interpretations not warranted from findings. | ***Low:*** Study design unclear or inappropriate choice of tools used. | ***Low:*** Unable to provide answers relatable to the review objectives. | ***Low:*** A must be accredited a 'low' in 2 domains to be excluded. |

## References


1. Institute of Medicine (US) Committee on Quality of Health Care in America. Crossing the Quality Chasm: A new healthcare system for the 21st century. Washington: National Academies Press (US); 2001.

2. Scottish Government. The Healthcare Quality Strategy for NHS Scotland. Edinburgh. Scottish Government; 2010.

3. Health Foundation. Overcoming challenges to improving quality. Lessons from the Health Foundation's improvement programme evaluations and relevant literature. London: Health Foundation; 2012.


For peer review only

4. Nursing and Midwifery Council. Standards for pre-registration nursing programmes. NMC. London; 2019.

5. Academy of Medical Royal Colleges. Quality improvement – training for better outcomes. Academy of Medical Royal Colleges. London; 2016.

6. QSEN Institute: Frances Payne Bolton School of Nursing. Quality and Safety Education for Nurses, 2010. Available at: 2010 (qsen.org)

7. Smith, F., Alexandersson, P., Bergman, B., Vaughn, L., Hellstrom, A. Fourteen years of quality improvement education in healthcare: a utilisation-focused evaluation using concept mapping. BMJ Open Quality, 2019. Available: https://doi.org: 10.1136/bmjoq-2019-000795.

8. Lillo-Crespo M, Sierras-Davó, M. C., MacRae, R., & Rooney, K. Developing a framework for evaluating the impact of Healthcare Improvement Science Education across Europe: a qualitative study. Journal of Educational Evaluation for Health Professions, 2017; 14(28). Available: https://doi.org/10.3352/jeehp.2017.14.28

9. Flynn, R., Scott, S.D., Rotter, T., Hartfield, D. The Potential for nurses to contribute to and lead improvement science in health care. Journal of Advanced Nursing, 2017; 73, p.97–107. Available: https://doi: 10.1111/jan.13164



For peer review only - http://bmjopen.bmj.com/site/about/guidelines.xhtml

10. Robb, G., Wells, S., Stolarek, I. and Bohm, G. The state of quality improvement and patient safety teaching in health professional education in New Zealand. New Zealand Medical Association, 2017; 130 (1464), p.13-24

11. Mak, V., Brand, G. and Morphet. J. Partnering with healthcare organisations to teach pre-registration health professions students about quality improvement: A systematic review. Journal of Clinical Nursing, 2022; p1-14.

12. Jones, A.C., Shipman, S.A., Ogrinc, G. Key characteristics of successful quality improvement curricula in physician education: a realist review. BMJ Quality Safety, 2014; 0, p.1-12.

13. Health Foundation. Complex Adaptive Systems. London: The Health Foundation; 2010.

14. Baillie, L., Bromley, B., Walker, M., Jones, R., Mhlanga, F. Implementing service improvement projects within pre-registration nursing education: A multi-method case study evaluation. Nurse Education in Practice, 2014, 14, p. 62-68.

15. Christiansen, A., Robson, L., Griffith-Evans, C., 2010. Creating an improvement culture for enhanced patient safety: service improvement learning in pre-registration education. Journal of Nursing Management. 18, p.782-788.



For peer review only - http://bmjopen.bmj.com/site/about/guidelines.xhtml

16. James et al. 2014 James, B., Beattie, M., Shepherd, A., Armstrong, L., Wilkinson, J., 2016. Time, fear and transformation: Student nurses' experiences of doing a practicum (quality improvement project) in practice. Nurse Education in Practice. 19, p.70-78.

17. Starr, S.R., Kautz, J.M., Sorita, A., Thompson, K.M., Reed, D.A., Porter, B.L., Mapes, D.L., Roberts, C.C., Kuo, D., Bora, P.R., Elraiyah, T.A., Murad, M.H., Ting, H.H. Quality Improvement Education for Health Professionals: A systematic review. American Journal of Medical Quality, 2016. 31(3), p.209-216.

18. Armstrong, L., Shepherd, A. & Harris, F.M. An evaluation of approaches used to teach quality improvement to preregistration healthcare professionals: an integrative review. International Journal of Nursing Studies, 2017; 73, p.70-84. https://doi.org/10.1016/j.ijnurstu.2017.05.005

19. Beaver, P. The challenges of safety improvement in New Zealand public hospitals, Kotuitui. New Zealand Journal of Social Sciences Online, 2019; 14(1), p112.125. Available at: https://doi.org/10.1080/1177083X.2018.1561476

20. Beal, J. Academic-Service Partnerships in Nursing: An Integrative Review, Nursing Research and Practice, 2012; p.1-9. Available at: https://doi:10.1155/2012/501564

For peer review only

21. Macphee, M. Developing a practice-academic partnership logic model. Nursing Outlook, 2009; 57, p.143-147.

22. Brown, D., White, J., Leibrandt, L. Collaborative partnerships for nursing faculties and health service providers: what can nursing learn from business literature? Journal of Nursing Management, 2006; 14, p.170–179

23. Nguyen, V.N.B, Lawerence, K., McGillion, A. The effectiveness of partnership models in clinical nursing education – A scoping review. Nurse Education Today, 2020; 90, p.1-8

24. Kaplan, C.H., Brady, P.W., Dritz, M.C., Hooper, D.K., Linam, M.W., Froehle, C.M. Margolis, P.M. The influence of context on quality improvement success in 20 healthcare: A systematic Review of the Literature. The Milbank Quarterly, 2010; 88 (4), p.500-599.

25. Health Foundation. Skilled for Improvement, Learning Communities and the Skills Needed to Improve Care and Evaluate Service Development. London: Health Foundation; 2014.

26. Pawson, R., Greenhalgh, T., Harvey, G. et al. Realist Synthesis: An Introduction. ESRC Research Methods Programme, University of Manchester; 2004.

For peer review only



For peer review only - http://bmjopen.bmj.com/site/about/guidelines.xhtml

27. Skivington, K., Matthews, L., Simpson, S.A., Craig, P., Baird, J., Blazeby, J.M., Boyd, K.A., Craig, N., French, D.P., McIntosh, E., Petticrew, M., Rycroft-Malone, J., White, M., Moore, L. A new framework for developing and evaluating complex interventions: update of Medical Research Council guidance. BMJ, 2021; 374. Available at: https://doi.org/10.1136/bmj.n2061

28. Windish, D.M., Reed, D.A., Boonyasai, R.T., Chakraborti, C., Bass, E.B. Methodological rigor of quality improvement curricula for physician trainees: A systematic review and recommendations for change. Academic Medicine, 2009; 84 (12), p.1677-1692. Available at: https://doi.org/10.1097/ACM.0b013e3181bfa080

29. Denzin, N.K. and Lincoln, Y.S. The sage handbook of Qualitative Research. London: Sage Publications Ltd; 2005

30. Creswell, J. W. Research Design: Qualitative, Quantitative and Mixed Methods Approaches. CA: Thousand Oaks Sage; 2014

31. Bates, R. A Critical analysis of evaluation practice: the Kirkpatrick model and the principle of beneficence. Evaluation and Program Planning, 2014; 27, p.341-347

32. Armstrong, L., Shepherd, A. and Harris, F. Putting QI education into context for pre-registration nurse education: an ethnographic multiple-case study. International Forum on Quality and Safety in Healthcare 2019. Tuesday 26-29th March, The Scottish Exhibition Conference Centre, Glasgow.



For peer review only - http://bmjopen.bmj.com/site/about/guidelines.xhtml

33. Coles, E., Anderson, J., Maxwell, M., Harris, F.M., Gray, N.M., Milner, G., MacGillivray, S. The influence of contextual factors on healthcare quality improvement initiatives: a realist review. Systematic Reviews. 2020, 26, 9(1)

34. Kaplan, H.C., Provost, L.P., Froehle, C.M., Margolis, P.A. The model for understanding success in quality (MUSIQ): building a theory of context in healthcare quality improvement. BMJ Quality and Safety, 2012; 21, p.13–20.

35. Bate, P. Context is everything. In: Perspectives on context. London: The Health Foundation; 2014. p. 1–29.

36. Greenhalgh, J. and Manzano, A. Understanding 'context' in realist evaluation and synthesis. International Journal of Social Research Methodology, 2021. Available at: https://doi.org/10.1080/13645579.2021.1918484

37. Tess, A., Vidyarthi, A., Yang, J., Myers, J.S. Bridging the Gap: A framework and strategies for integrating the quality and safety mission of teaching hospitals and graduate medical education. Academic Medicine, 2015; 90 (9), p. 1251-1257.

38. Moher, D., Liberati, A., Tetzlaff, J., Altman, D.G., Prisma Group. Preferred reporting items for systematic reviews and meta-analyses: the PRISMA statement. Open Medicine, 2009; 3(2), p123-130



For peer review only - http://bmjopen.bmj.com/site/about/guidelines.xhtml

39. Wong, G., Greenhalgh, T., Westthorp, G., Buckingham, J., Pawson, R. RAMESES publication standards: realist syntheses. BMC Medicine, 2013; p.11-21. Available at: https://doi.org/10.1186/1741-7015-11-21

40. Abrams, R., Park, S., Wong, G. Rastogi, J, Boylan, A.M., Tierney, S., Petrova, M., Dawson, S., Roberts, N. Lost in reviews: Looking for the involvement of stakeholders, patients, public and other non-researcher contributors in realist reviews, 2020; 12(2), p. 239-247.

41. Pawson, R., Greenhalgh, T., Harvey, G., Walshe, K. Realist review—a new method of systematic review designed for complex policy interventions. Journal of Health Services Research and Policy, 2005; 10 p.21–34.

42. Ogrinc, G., Armstrong, G., Dolansky, M.A., Singh, M.T., Davies, L. SQUIRE-EDU (Standards for Quality Improvement Reporting Excellence in Education): Publication Guidelines for Educational Improvement. Academic Medicine, 2019; 94(10), p.1461-1470. Available at: doi: 10.1097/ACM.0000000000002750.

43. Gough, D. Weight of Evidence: A Framework for the appraisal of the quality and relevance of evidence, Research Papers in Education, 2007, pp. 213-228.



For peer review only - http://bmjopen.bmj.com/site/about/guidelines.xhtml

For peer review only



For peer review only - http://bmjopen.bmj.com/site/about/guidelines.xhtml

# PRISMA-P 2015 Checklist

**This checklist has been adapted for use with systematic review protocol submissions to BioMed Central journals from Table 3 in Moher D et al:** Preferred reporting items for systematic review and meta-analysis protocols (PRISMA-P) 2015 statement. *Systematic Reviews* 2015 **4**:1

An Editorial from the Editors-in-Chief of *Systematic Reviews* details why this checklist was adapted - **Moher D, Stewart L & Shekelle P**: Implementing PRISMA-P: recommendations for prospective authors. *Systematic Reviews* 2016 **5**:15

| Section/topic | # | Checklist item | Information reported | | Line number(s) |
|---|---|---|---|---|---|
| | | | Yes | No | |
| **ADMINISTRATIVE INFORMATION** | | | | | |
| **Title** | | | | | |
| Identification | 1a | Identify the report as a protocol of a systematic review | | Realist Review | Page 1<br>Line 3 |
| Update | 1b | If the protocol is for an update of a previous systematic review, identify as such | | No | |
| **Registration** | 2 | If registered, provide the name of the registry (e.g., PROSPERO) and registration number in the Abstract | Yes | | Page 3<br>Lines 4-5 |
| **Authors** | | | | | |
| Contact | 3a | Provide name, institutional affiliation, and e-mail address of all protocol authors; provide physical mailing address of corresponding author | Yes | | Page 1<br>Lines 1-28 |
| Contributions | 3b | Describe contributions of protocol authors and identify the guarantor of the review | Yes | | Page 17<br>Lines ?? |
| **Amendments** | 4 | If the protocol represents an amendment of a previously completed or published protocol, identify as such and list changes; otherwise, state plan for documenting important protocol amendments | | No | |
| **Support** | | | | | |
| Sources | 5a | Indicate sources of financial or other support for the review | Yes | | Page 17<br>Lines ?? |

For peer review only

2

| Section/topic | # | Checklist item | Information reported Yes | No | Line number(s) |
|---|---|---|---|---|---|
| Sponsor | 5b | Provide name for the review funder and/or sponsor | | | Page 17<br>Lines ?? |
| Role of sponsor/funder | 5c | Describe roles of funder(s), sponsor(s), and/or institution(s), if any, in developing the protocol | | No funders | |
| **INTRODUCTION** | | | | | |
| **Rationale** | 6 | Describe the rationale for the review in the context of what is already known | Yes | | Page1<br>Lines 31-34<br>Page2<br>Lines 1-9 |
| **Objectives** | 7 | Provide an explicit statement of the question(s) the review will address with reference to participants, interventions, comparators, and outcomes (PICO) | | This is not appropriate for Realist Review methodology | |
| **METHODS** | | | | | |
| **Eligibility criteria** | 8 | Specify the study characteristics (e.g., PICO, study design, setting, time frame) and report characteristics (e.g., years considered, language, publication status) to be used as criteria for eligibility for the review | | This is not appropriate for Realist Review methodology | |
| **Information sources** | 9 | Describe all intended information sources (e.g., electronic databases, contact with study authors, trial registers, or other grey literature sources) with planned dates of coverage | Yes | | Page 12<br>Line 9-20 |
| **Search strategy** | 10 | Present draft of search strategy to be used for at least one electronic database, including planned limits, such that it could be repeated | Yes | | Page 19<br>Lines 1-37 |
| ***STUDY RECORDS*** | | | | | |

BioMed Central
The Open Access Publisher

| Section/topic | # | Checklist item | Information reported: Yes | Information reported: No | Line number(s) |
|---|---|---|---|---|---|
| Data management | 11a | Describe the mechanism(s) that will be used to manage records and data throughout the review | Yes | | Page 13<br>Lines 12-26 |
| Selection process | 11b | State the process that will be used for selecting studies (e.g., two independent reviewers) through each phase of the review (i.e., screening, eligibility, and inclusion in meta-analysis) | Yes | | Page 13-14<br>Lines 20-28<br>Lines 1-4 |
| Data collection process | 11c | Describe planned method of extracting data from reports (e.g., piloting forms, done independently, in duplicate), any processes for obtaining and confirming data from investigators | Yes | | Page 14<br>Lines 5-16 |
| **Data items** | 12 | List and define all variables for which data will be sought (e.g., PICO items, funding sources), any pre-planned data assumptions and simplifications | | This is not appropriate for Realist Review methodology | |
| **Outcomes and prioritization** | 13 | List and define all outcomes for which data will be sought, including prioritization of main and additional outcomes, with rationale | Yes | | Page 15<br>Lines 2-24 |
| **Risk of bias in individual studies** | 14 | Describe anticipated methods for assessing risk of bias of individual studies, including whether this will be done at the outcome or study level, or both; state how this information will be used in data synthesis | Yes | | Pages 14-15<br>Lines 17-28 |
| ***DATA*** | | | | | |
| **Synthesis** | 15a | Describe criteria under which study data will be quantitatively synthesized | | This is not appropriate for Realist Review methodology | |
| | 15b | If data are appropriate for quantitative synthesis, describe planned summary measures, methods of handling data, and methods of combining data from studies, including any planned exploration of consistency (e.g., $I^2$, Kendall's tau) | Yes | | Page 15<br>Lines 2-24 |
| | 15c | Describe any proposed additional analyses (e.g., sensitivity or subgroup analyses, meta- | | | n/a |

BioMed Central The Open Access Publisher

4

| Section/topic | # | Checklist item | Information reported Yes | Information reported No | Line number(s) |
|---|---|---|---|---|---|
| | | regression) | | | |
| | 15d | If quantitative synthesis is not appropriate, describe the type of summary planned | | | Page 15<br>Lines 21-24 |
| **Meta-bias(es)** | 16 | Specify any planned assessment of meta-bias(es) (e.g., publication bias across studies, selective reporting within studies) | | | n/a |
| **Confidence in cumulative evidence** | 17 | Describe how the strength of the body of evidence will be assessed (e.g., GRADE) | Yes | | Page 15<br>Lines 17-28 |

For peer review only



BioMed Central
The Open Access Publisher

BMJ Open

# How, and under what contexts, do academic-practice partnerships collaborate to implement healthcare improvement education into preregistration nursing curriculums: a realist review protocol.

| | |
|---|---|
| Journal: | *BMJ Open* |
| Manuscript ID | bmjopen-2023-077784.R1 |
| Article Type: | Protocol |
| Date Submitted by the Author: | 22-Sep-2023 |
| Complete List of Authors: | Armstrong , Lorraine ; University of Stirling, Sport and Health Sciences<br>Moir, Chris; University of Otago, Centre for Post Graduate Nursing Studies<br>Taylor, Peta; Ara Institute of Canterbury Ltd, Department of Health Practice |
| <b>Primary Subject Heading</b>: | Nursing |
| Secondary Subject Heading: | Nursing |
| Keywords: | Health Education, Systematic Review, Nursing Care, Quality in health care < HEALTH SERVICES ADMINISTRATION & MANAGEMENT |
| | |

SCHOLARONE™
Manuscripts

**Title**: How, and under what contexts, do academic-practice partnerships collaborate to implement healthcare improvement education into preregistration nursing curriculums: a realist review protocol.

Corresponding Author:

**Mrs Lorraine Armstrong RN (A), (BSc).**

Lecturer in Nursing

School of Health Sciences

University of Stirling

Scotland

FK9 4LA

Tel: 01786 466117

Email: lorraine.armstrong@stir.ac.uk

ORCHID 0000-0001-8951-2712

**Dr Chris Moir RN, (PhD).**

Clinical co-ordinator/Senior Lecturer

Centre for Post Graduate Nursing Studies

University of Otago

8140 Christchurch

New Zealand

Tel: +64 3 364 3869

Email: chris.moir@otago.ac.nz

ORCHID 0000-0002-9178-2540

**Dr Peta Taylor RN, (PhD).**

Senior Nursing Lecturer

For peer review only



For peer review only - http://bmjopen.bmj.com/site/about/guidelines.xhtml

Department of Health Practice
Ara Institute of Canterbury Ltd
8140 Christchurch
New Zealand
Tel: +64 3 940 7556
Email: peta.taylor@ara.ac.nz
ORCID 0000-0002-9182-8254

## Abstract

**Introduction**: Internationally, healthcare improvement remains a clinical and educational priority. Consensus in Europe, Canada and the USA to implement quality improvement (QI) education into preregistration nursing curricula ensures students become equipped with the skills and knowledge required to improve practice. Now, New Zealand and Australia are beginning to implement QI education into their nursing curricula. However, QI education is complex; comprising multiple components, each influenced by the contexts under which they are developed and implemented. Evaluation studies of QI education unanimously acknowledge that academic and practice partnerships (APPs) are essential to optimally embed QI into preregistration curricula, yet it is not understood how, and under what contexts, APPs collaborate to achieve this.

**Methods and analysis**: A realist review to determine how, and under what contexts, APPs collaborate to implement QI education in pre-registration nursing will be conducted using the RAMSES Guidelines. International stakeholders will be consulted at each stage which includes (1) clarifying the scope of the review through empirical literature and tacit expert knowledge, (2) searching for evidence in healthcare and social science databases/grey literature, (3) appraising studies using the EPPI weight of evidence framework and extracting data using SQUIRE-EDU Publication Guidelines, (4) synthesising evidence and drawing



For peer review only

For peer review only - http://bmjopen.bmj.com/site/about/guidelines.xhtml

conclusions through the creation of context, mechanism and outcome configurations and (5) disseminating findings through conferences and peer-reviewed publications.

**Ethics and Dissemination**: Ethical approval was not required for this study. Findings will be disseminated to international nurse educators, leaders and front-line staff implementing QI education within their own academic and practice contexts through conferences and peer-reviewed publications.

**Prospero Registration Number**

CRD42021282424

**Keywords**

Realist Review, Pre-registration Nursing Education, Academic-practice Partnerships, Context, Quality Improvement

**Strengths and limitations of this study**

• Realist review is novel to explore QI education in preregistration nursing.

• Understanding academic-practice partnerships in developing QI education is novel.

• Focus on the influence of context on QI education builds on the current literature.

• A limitation of the study is that only English articles will be included.

# Introduction

## Healthcare improvement in nurse education

This millennium has witnessed a surge in healthcare improvement practice and educational development in response to practice errors and increased risk to patients' safety (1). Therefore, developing workforce capacity and capability in improvement knowledge, skills and behaviours remains a priority on policy and educational agendas internationally (2-6). In

For peer review only

the past decade, countries such as the United Kingdom (UK), Sweden, Spain, Canada and USA have developed consensus that QI education in preregistration nursing curricula should be a regular and ongoing process, if students are to contribute to and lead practice improvements (4, 6-9). Recently, New Zealand has begun to embark on QI curricular integration, having established from 49 educational institutions that few display the foundations of quality and safety education (10). It is recognised that failure to address this gap compromises graduate nurses' abilities to implement and sustain healthcare improvement. A similar drive for QI education in pre-registration nursing curricula has also emerged from Australia where much of the focus is around partnership working (11). The authors of this review protocol have integrated, or are about to integrate, QI curricula into their nurse education programmes in the UK and New Zealand and have a shared purpose to develop valuable insights into how APPs collaborate to optimally embed QI into nurse education.

To date QI educational content in preregistration nursing has included improvement methods, quality indicator measures, the Model for Improvement, Plan-Do-Study-Act cycles, root cause analysis, systems thinking, inter-professional learning, clinical governance, data, human factors and evidence-based practice, which, when delivered through an experiential learning pedagogical approach, is regarded most appropriate to equip pre-registration students with the necessary skills and knowledge about QI. Supplementary approaches include didactic learning, seminars, group work, self-directed learning and/or simulation (12).

QI education is regarded as a complex social phenomenon, whereby its numerous components such as the learner, the curricula, the faculty and the practice setting all interact on multiple levels of the system. They are also subject to the varying contexts under which they are developed and implemented (13). Considering QI education from a *'systems thinking'* perspective is useful here, because this approach views the components as a connected whole rather than separate component parts (14).

There are many component parts which contribute to the complex nature of developing and implementing preregistration QI nurse education. The literature describes



For peer review only - http://bmjopen.bmj.com/site/about/guidelines.xhtml

these as: developing the curricula, delivering teaching content, creating real life practice-based QI opportunities, developing staff expertise in improvement methodologies, securing mentorship and support, bridging support through information provision and link staff, updating student mentors on QI education through workshops, developing resource handbooks, seeking approval of students' improvement ideas, access to data, governance of ethical student practice, creating a QI culture where practice staff facilitate and mediate between students and other staff members, ongoing development and evaluation of QI education, and lastly supporting dissemination activities to showcase students QI work (14-18). Taking account of these multiple components, it is evident that developing and implementing complex QI educational curricula requires synchronised planning between academics and practitioners, if students are to engage in meaningful practice-based improvement opportunities.

Despite a growing interest emerging from academics and practitioners to implement QI nurse education, there is currently no research to inform how, and under what contexts, APPs collaborate to achieve this (10-11, 19).

<u>Academic-practice partnerships</u>

Academic-practice partnerships (APPs) are defined as '*strategic associations between educational and clinical facilities that are established to advance their mutual interests in nursing practice, education and research'* (20). Their purpose ranges from developing preceptorship education to clinical nurse leadership initiatives in hospitals, community health and public health agencies, nursing homes, schools, and governmental agencies (20-21). They operate on strategic, tactical, operational, interpersonal and cultural levels (Brown 22).

There has been a recent review that considered the effectiveness of nurse APPs on costs, employability, work-readiness, confidence, competence and stakeholder satisfaction, but not in developing or implementing nurse education (23). The same review also encourages future research to focus on *how* APPs function and sustain under different



For peer review only - http://bmjopen.bmj.com/site/about/guidelines.xhtml

contexts. As such, how APPs function and sustain under different contexts when developing and implementing QI nurse education remains our focus.

We do know though that nursing APPs have been investigated in the management literature which attribute several key principles to successful collaboration (22). These include creating a shared vision, establishing accountability, nurturing appropriate people engagement with balanced relationships, maintaining trust and respect, practising good communication and putting governance in place. Through these principles, APPs benefit from improved organisational efficiencies, enhanced opportunities for innovation and enhanced recruitment and retention (22).

Similarly, there is evidence from the management literature of investigations into how different types of partners in healthcare improvement practice and research, not education, generally work together (24). It is suggested that healthcare improvement partners require three types of skills to function and be sustained. These relate to technical skills (Lean, PDSA and run charts), soft skills (communication, leadership, team skills) and learning skills (knowledge sharing, encouraging participation and collective learning). In the absence of one skill subset, it is reported that healthcare improvement partners experience difficulties leading change (25).

Lastly, a scoping review collating evidence of the effectiveness of partnerships in clinical nurse education has also been undertaken; however, the eligible studies in this review adopt weak evaluation measures and use low-quality appraisal instruments to determine overall effectiveness (23).

If nursing APPs are to develop and implement QI education under their own practice and education contexts, then insights into the experiences of this process is essential.

<u>What will a realist review add to the field?</u>

Realist methodology is a theory-driven approach appropriate for investigating how APPs work together, because it not only considers the complex nature of QI education which



For peer review only - http://bmjopen.bmj.com/site/about/guidelines.xhtml

operates as a social intervention but recognises the different contexts and mechanisms that contribute or not to the QI educational programme's success (26). The Medical Research Council (MRC) have recently updated their complex intervention guidelines to encourage realist approaches for evaluation studies (27).

A previous review that has investigated the effectiveness of APPs working in clinical education, and one examining the methodological rigour in QI curricula, both criticise studies for the research approaches they have adopted (23, 28). This is because, predominantly, researchers investigating QI education have asserted a positivist stance. Traditionally, positivists sit within the natural (physical) sciences, are associated with quantitative approaches and claim that an absolute truth of knowledge exists (29).

Realist researchers also sit within the natural (physical) sciences as post-positivists but challenge an absolute truth theory in favour of a realist perspective. Both sides proclaim broadly similar *effect* and *outcome* orientations, but post-positivists argue that only an approximation of truth exists, and as such focus on the mechanistic factors which determine outcomes, as opposed to the outcomes themselves (30).

Positivist stances and quantitative approaches are regarded as too simple to determine causal linkages between complex interventions and outcomes because they discount the all-important concept of context (31). Ignoring context is considered to be the antithesis of healthcare improvement principles and is instead a pre-requisite to understanding why success ensues in one instance and not in another. The context debate is infinitely evolving, and in-depth discussions are widely available (24-25). However, in general terms it relates to *everything*. Context has become the central focus of QI nurse education studies recently, in which ethnographic methodology is used to explore the contexts that influence student nurses QI learning experiences in the practice setting (32).

There have been many frameworks developed to study context in healthcare improvement (33). For instance, The Model for Understanding Success in Quality improvement (MUSIQ) targets contexts relating to micro-systems; these include the QI team, QI support and capacity, the organisation, and the external environment (34) while others



For peer review only - http://bmjopen.bmj.com/site/about/guidelines.xhtml

consider context as the *outer* components (political, social and cultural) and *inner* components (organisational structure, size and performance) (35). In terms of realist reviews, Greenhalgh and Manzano (36) have more recently identified context as (1) tangible, fixed, observable features that trigger mechanisms or (2) relational and dynamic features that shape the mechanisms through which the intervention works.

For the purpose of exploring '*contexts'* this review protocol will draw upon Tess et al.'s conceptual framework of integrating quality and safety into medical education (37). We did not come across a similar model which exists for nurse education in the literature. The concepts in their model will form our preliminary investigation into the contexts APPs might operate under. The contexts we are initially guided towards, but will not be restricted by, are organisational culture, academic-practice alignment, infrastructure, curricular resources, faculty-practice development and inter-professional collaboration (see Figure 1). As our review progresses and our data evolves, we will develop context themes iteratively and report upon any further developments in the final write up of the study.

- Insert Figure 1 here

## Methods and analysis

For completeness and transparency this review protocol will uphold the recommendations of PRISMA-P (Preferred reporting items for systematic review and meta-analysis protocols), where it relates to our realist review design and is available in additional file one (38). The synthesis process of this study protocol commenced in June 2020 and is predicted to conclude in June 2024.

<u>Patient and Public Involvement</u>

Patients and public were not involved in the development of the realist review protocol.



For peer review only - http://bmjopen.bmj.com/site/about/guidelines.xhtml

Study Design

Our realist review of the literature will be guided and conducted in line with the Realist and Meta-narrative Evidence Syntheses: Evolving Standards (RAMSES) Guidelines (39). This approach will enable a rigorous process to be followed ensuring that both methodological decisions remain appropriate to the review type and anticipated data set. The steps are (1) clarifying the scope of the review through empirical literature and the authors' tacit expert knowledge, (2) searching for evidence in healthcare and social science databases, and grey literature, (3) appraising studies using the Evidence for Policy and Practice Information and Co-ordinating Centre (EPPI) weight of evidence framework and extract data using SQUIRE-EDU (Standards for QUality Improvement Reporting Excellence in Education): Publication Guidelines, (4) synthesising evidence and drawing conclusions through the creation of context, mechanism and outcome configurations and (5) disseminating findings through conference presentations and peer-reviewed publications.

Study Methods

**Clarifying the scope**

The original scope of our realist research stems from the findings of our previous research. Firstly, an integrative review which explored QI education in pre-registration healthcare examined QI educational interventions in pre-registration healthcare. This study concluded that there was a need to develop and strengthen APPs to embed QI in nurse education (12). Equally, a drive from New Zealand and Australian healthcare sectors to embed QI into their nursing programmes has broadened the scope of the review and created an opportunistic and timely collaboration to develop insights into how APPs have implemented QI education internationally (10-11, 19) This evolving landscape, coupled by the authors' academic and clinical tacit knowledge of developing and implementing QI nurse education, has shaped the initial aim and objectives early in the process.

*Study Aim*

The aim of our realist review is to develop an understanding of *'how'* and under what contexts APPs collaborate to implement healthcare improvement education into pre-registration nursing curriculums.

*Study Objectives:*

The key objectives of the research review are to:

1. Identify the contexts under which APPs collaborate to implement QI into preregistration nurse education
2. Analyse the mechanisms and outcomes associated with each context under which APPs collaborate to implement QI into preregistration nurse education
3. Explore relationships emerging across and between the contexts, mechanisms and outcomes in which APPs collaborate to implement QI into preregistration nurse education
4. Explain the causal pathways of how APPs collaborate to implement QI into preregistration nurse education
5. Disseminate findings so that nursing and academic institutions, new to QI education, are encouraged and well informed to develop their own highly effective APPs

The next step entailed undertaking a broader exploration of the literature to enable us to refine the review's parameters. We identified a key difference between international countries; that being some healthcare systems such as the UK, New Zealand, Australia and Canada, were publicly funded compared to that of the USA which is privately funded. Exploring both healthcare systems was believed to be too vast for the team's time and resource, however our scoping activity retrieved lower-than-expected numbers of studies from outside of the USA, who remain international leaders in healthcare improvement education and research. As a result, we broadened the review's international range and will contemplate whether this difference in healthcare systems could act as a potential '*context'* category under which APPs collaborate.

For peer review



For peer review only - http://bmjopen.bmj.com/site/about/guidelines.xhtml

Lastly, the core research team held a stakeholder engagement exercise to assist in the development of the study in December 2021. Stakeholders that were contacted included international academic and practice staff operating on multiple levels of the healthcare and academic organisations such as pre and post registration nurses, nurse directors, nurse academics, clinical nurse educators, nursing directors of quality and nursing journal editors. We provided an opportunity to shape the realist review protocol, make adaptations to the study methods, discuss pressing issues, and then actioned suggestions to refine the scope of the review. Stakeholders are listed in the acknowledgements. Consideration of the value that patients, carers and the public add to the project will be regularly reviewed by the team, acted upon for input where applicable and reported in the final review manuscript (40).

**Developing our programme theory**

Programme theories in realist review relate to the intervention under investigation, in this case the QI educational programme. They seek to offer a theoretical explanation of how the intervention works, for whom, and under what contexts. Seeking out assumptions of how interventions operate under different contexts, through a variety of mechanisms which produce different outcomes, is a continuous, collaborative and an iterative process which uses theoretical and empirical literature to populate and test initial '*rough theories'* (39). A number of initial rough theories about how APPs collaborate to implement healthcare improvement education have influenced our investigation:

- Experiential learning is the most appropriate pedagogical approach to equip student nurses with adequate QI knowledge and skills. Therefore, we anticipate that early formation of APPs is needed to streamline quality teaching and learning experiences.

- The international landscape varies, and some APPs are more experienced at implementing QI education than others. It is possible that policy drivers and/or



For peer review only - http://bmjopen.bmj.com/site/about/guidelines.xhtml

professional standards from within each country will determine to a greater/lesser extent, the priority by which QI education is implemented.

- APPs operate on multiple levels of the organisation just as QI education comprises multi components that requires a '*systems thinking'* perspective. We predict that multiple APPs may operate simultaneously, over multiple levels of the organisation to implement one QI education programme, rather than one APP operating on an individual basis.

In line with realist review methods, our '*initial rough theories'* stated above, will continue to be refined and/or expanded in an iterative manner as we explore further literatures, and analyse and populate our data into our context framework (39).

**Search for evidence**

Approaches to evidence searching in realist reviews are diverse and dependent on the discipline and study objectives; on average three to four iterative phases are undertaken (41). Our protocol remains at the earliest phase of our evidence search after which we will undertake further phases before completion.

Atypically, we employed traditional systematic searching for empirical literature early to complement our scoping review. Whilst this method is typically classed as the 'main' search in realist reviews (41), our familiarity with the dearth of existing research in this discipline, led us to plan our 'main' evidence search within the unpublished literature, internationally. Therefore, as an adjunct to our scoping review we undertook early empirical searching of QI education within social science and healthcare databases.

*Electronic databases*

Electronic databases: Cumulative Index to Nursing and Allied Health Literature (CINAHL), EMcare, Medical Literature Analysis and Retrieval System Online (MEDLINE), Science Direct and Scopus were systematically searched. Studies were limited to peer-reviewed and



For peer review only - http://bmjopen.bmj.com/site/about/guidelines.xhtml

English language studies. Aligning to the inception of QI nurse education in the millennium, databases were searched from 2000-2021. A primary search for MEDLINE was developed using MeSH (Medical Subject Headings) terms. Subsequent searches were translated to each database through Boolean terms, truncation, wildcards and proximity searching to maximise relevant papers. A primary sample search strategy from MEDLINE is presented (see Table 1).

In the next phase, our 'main' evidence search will involve locating international policy documents, online blogs, professional body documentation, healthcare and educational establishments' websites, social media, dissertations, opinion articles, editorials and google scholar. We will gather evidence to support or refute our programme theories. Search terms will draw upon the MEDLINE strategy as well as focusing on policy, management, governance, workforce, system thinking and healthcare education.

Finally, we will update previous search strategies and present evidence to our stakeholders for their comments. We will seek the opportunity to ask them to signpost us to potentially eligible evidence. Once saturation is deemed to be reached, results yielded from each phase will be transparent and illustrated in a flow diagram (42).

*Reference list searching*

As with current evidence, all future evidence will undergo scanning of reference lists to identify eligible evidence not detected initially. Where difficulty in retrieving evidence occurs, authors or organisations will be contacted to request the evidence.

**Inclusion/Exclusion Criteria**

*Inclusion*

Empirical searches, both current and future, will include international peer-reviewed studies of all designs where abstracts are written in English from 2000 through to 2021 and respectively 2023 when searches are rerun. Studies will need to describe an element of



For peer review only - http://bmjopen.bmj.com/site/about/guidelines.xhtml

APPs collaboration to implement QI education in preregistration nursing. We accept '*quality improvement education*' where it describes the teaching, learning or utility of a healthcare improvement model and details a theory and practice-based element.

*Exclusion*

Current and future empirical evidence will exclude studies which make no reference to how the APPs collaborated, refer solely to online learning or patient safety content, or do not include preregistration nursing students.

- Insert Table 1 here

**Data Collection and Management**

*Search for evidence*

For all current and future evidence, reviewers (LA, CM and PT) will conduct and save searches within corresponding databases where applicable. To ensure each search remains accessible, reproducible and transparent a manual record keeping log will continue to be produced. To allow reviewers access, studies eligible for screening will be uploaded electronically to a shared named folder within Microsoft Teams. Duplicates will be removed.

*Screening and selection of studies*

Screening and selection of current and future evidence will follow the same process by which reviewers (LA, CM and PT) will work independently to screen titles and abstracts and/or executive summaries from retrieved evidence using predetermined inclusion and exclusion criteria. Ambiguities around papers will be discussed and the decision to include or exclude them will be made jointly. Evidence for inclusion will continue to be transferred to a separate shared named folder to allow reviewer access for data extraction. Some excluded evidence may remain valuable for later discussion of the review findings, as such these will be stored in a separate share folder in Microsoft Teams. To ensure consistency in the application of inclusion and exclusion criteria, one reviewer with subject expertise (LA) will



For peer review only - http://bmjopen.bmj.com/site/about/guidelines.xhtml

review a 10% random sample of the evidence to confirm inter-rater reliability. Discrepancies will be resolved by discussion.

*Data extraction & appraising the evidence*

Reviewers (LA, CM and PT) will extract data with cognisance of SQUIRE-EDU (Standards for QUality Improvement Reporting Excellence in Education): Publication Guidelines for Educational Improvement (43). Reviewers will also focus on extracting data under the headings which relate to the key *'contexts'* of interest from Tess et al.'s framework (37). These include the organisational culture, practice-academic alignment, infrastructure, curricular resources, faculty-practice development and inter-professional collaboration. Mechanisms relating to each *'context'* will be extracted and included under each heading. Reviewers will also extract the outcomes of interest that are detailed by authors in each study. One reviewer with expert subject knowledge (LA) will compare a 10% random sample to ensure consistency. Discrepancies will be resolved by discussion or a fourth reviewer where necessary.

A realist review is predicted to retrieve heterogeneity in study design, therefore using a singular hierarchical appraisal tool for exclusion will not be fit for purpose or add value to answering the study's questions. The EPPI Weight of Evidence framework which considers the use of judgement in relevance and quality will be used (Table 2). This framework allocates each study with a weight of high, medium or low in relation to three key areas: (a) Trustworthiness of results of study, (b) appropriateness of study design to the review question and (c) appropriateness of focus to answering the review question. The results of (a), (b) and (c) are combined and given an overall weight. This method allows the worth of each study to be identified for the realist synthesis rather than being a preliminary pre-qualification exercise (44). To ensure consistency in overall Weight of Evidence allocated, reviewers will meet to compare a 10% random sample of low, medium and high graded papers and discrepancies resolved through discussion or a fourth reviewer if necessary.

- Insert Table 2 here



For peer review only - http://bmjopen.bmj.com/site/about/guidelines.xhtml

*Analysis and synthesis of evidence*

As a general framework, the Realist and Meta-narrative Evidence Syntheses: Evolving Standards (RAMSES) Guidelines will be applied (39). Analysis in realist reviews is primarily concerned with refining theory-driven explanations, in this case, of how APPs work collaboratively to implement QI education under different contexts. Analysis will involve, firstly: identifying contexts under which APPs collaborate and informed but not limited by a preliminary framework of QI education developed by Tess et al. (37). We will discuss new emerging contexts and consider what supporting evidence warrants its inclusion to refine our programme theories. In line with *systems thinking,* we will explore the relationships that exist as a collective whole, in order to create a broader perspective of QI education as a holistic system and add depth to our understandings.

Next, the mechanisms and outcomes associated with each context will be investigated and listed as context, mechanism and outcome (CMO) configurations. A CMO configuration '*is a statement, diagram or drawing that spells out the relationship between particular features of context, particular mechanisms and particular outcomes'* (39). The causal relationships within each CMO configuration will be explored and listed. Recurring themes will be identified and grouped as demi-regularities (semi-predictable patterns). We will consider how the evidence supports or disproves our initial rough theories of how APPs collaborate to implement QI education into preregistration nurse education. The findings will be presented as a narrative synthesis which the contexts and any relationships between them will be discussed. The demi-regularities associated with each context will be embedded into this discussion as we see appropriate, and our conclusions will take account of the current literature landscape to make future recommendations.

*Disseminate, implement and evaluate*

The research will be disseminated through published works, local and international conference presentations. The findings will be used by two New Zealand tertiary institutions and their partnering district health boards to develop effective APPs to implement QI education in their nursing programmes. A realist process evaluation will be undertaken to



For peer review only - http://bmjopen.bmj.com/site/about/guidelines.xhtml

advance the field of QI education and continue knowledge development around APPs working.

## Ethics and dissemination

No ethical approval was required to undertake this study. This realist review is the first to our knowledge that aims to develop an understanding around how, and under what contexts, APPs collaborate to implement QI education in pre-registration nursing curriculums. This study is aimed at international nurse educators and nurse leaders who can make sense of the findings to adapt their implementation approach within the limits of their own education and practice contexts. We will disseminate findings through conference presentations and peer-reviewed journals.

*Author Statement*

LA, CM and PT made substantial contributions to the conception and design of the protocol development. All authors have approved the submitted version and have agreed to be personally accountable for own contributions and ensure questions related to the accuracy or integrity of the work, even ones in which the author was not personally involved, are appropriately investigated, resolved, and the resolution documented in the literature.

*Funding statement*

This research received no specific grant funding from any funding agency in the public, commercial or not-for-profit sectors.

*Competing interests*

The authors declare that they have no competing interests.



For peer review only - http://bmjopen.bmj.com/site/about/guidelines.xhtml

*Word count*

4077

*Acknowledgements*

We would like to acknowledge the following stakeholders who contributed to the development of this realist review protocol.

- Jen Gow (RN) Nurse Consultant for Quality and Performance Improvement: Southern District Health Board, New Zealand
- Verity Mak (Med, MN, BN, Lecturer): Monash University, Victoria, Australia
- Professor Maura McPhee (RN, PhD, FCNEI): University of British Columbia, Canada
- Henry Ritche (RN, MNSc): Wellington Regional Hospital, New Zealand
- Angela Williams (Mrs.) Senior Lecturer in Nursing (Adult): Wrexham Glyndwr University, Wales, United Kingdom
- Susan Wood (RGON BSc AdvN MNS FCNA, Director of Quality and Patient Safety): Te Whatu Ora Waitaha Canterbury and Te Tai o Poutini West Coast New Zealand

*Data Statement*

The datasets used and/or analysed during the current study are available from the corresponding author on reasonable request.



For peer review only - http://bmjopen.bmj.com/site/about/guidelines.xhtml

Table 1. Developed Search Strategy for MEDLINE using OVID

1. Quality improvement/
2. Quality improvement.tw.
3. Quality Assurance, Health care/
4. Quality assurance.tw.
5. improvement science.tw.
6. improvement methodology*.tw.
7. Improvement model*.tw.
8. service improvement.tw.
9. CQI.tw.
10. plan do study act.tw.
11. PDSA.tw.
12. Plan do check act.tw.
13. PDCA.tw.
14. Total Quality Management/
15. Total quality management.tw.
16. TQM.tw.
17. 1 or 2 or 3 or 4 or 5 or 6 or 7 or 8 or 9 or 10 or 11 or 12 or 13 or 1 or 14 or 15 or 16
18. Interinstitutional Relations/
19. Interinstitutional relations*.tw.
20. Public-Private Sector Partnerships/
21. Public-Private partner*.tw.
22. academic practice partner*.tw.
23. intersectoral collaboration?.mp (mp=title, abstract, original title, name of substance word, subject, heading word, floating sub-heading word, keyword heading word, organism supplementary concept word, protocol supplementary concept word, rate disease supplementary concept word, unique identifier, synonyms)



For peer review only - http://bmjopen.bmj.com/site/about/guidelines.xhtml

24. collaborat*.tw.

25. communit* of practice.tw.

26. 18 or 19 or 20 or 21 or 22 or 23 or 24 or 25

27. Health Personnel/ed (Education)

28. Health personnel education.tw.

29. Health staff education.tw.

30. Exp Education, Nursing/

31. Nursing education.tw.

32. allied health education.tw.

33. or 28 or 29 or 30 or 31 or 32

34. 17 and 26 and 33

35. Limit 34 to (English language and yr="2000-Current")

36. From 35 keep

1, 12, 14, 28, 37, 43, 57, 66, 71, 74, 81, 94-96, 102-103, 117, 123, 128, 130-131, 136, 140, 144, 147, 149, 151, 163, 169, 174, 198, 219

For peer review only

Table 2. EPPI Weight of Evidence

| | **WoE A** | **WoE B** | **WoE C** | **Overall WoE** |
|---|---|---|---|---|
| **Weight of Evidence** | **Trustworthiness of results of study** | **Appropriateness of study design to review question** | **Relevance of study focus to answer review question** | **A,B & C Combined** |
| | ***High*:**<br>**1.Intervention clearly articulated (who, what when, where, why) to allow for reproducibility.**<br>**2.Ethical, explicit and detailed methods sections for recruitment, data collection & analysis.**<br>**3.Comprehensive reporting of data and accurate interpretation clearly warranted from findings.** | ***High*:**<br>**Large-scale multi-method designs e.g., RCT's, quasi-experimental using validated tools and/or interview, observational data with large sample size.** | ***High*:**<br>**Provides useful & relevant answers closely relatable to review objectives.** | ***High*:**<br>**A must be accredited no less than a 'high' in two domains to achieve an overall WoE of High.** |
| | ***Medium*:**<br>**1.Intervention described but not with sufficient detail to replicate.**<br>**2.Satisfactory reporting of methods sections for ethics, recruitment, data collection & analysis - minor omissions evident.**<br>**3.Minor bias in reporting of data and interpretations partially warranted from findings.** | ***Medium*:**<br>**Medium-scale qualitative, quantitative or mixed method designs e.g., local pre-post, post survey/questionnaire using acceptable tools and/or interview, observational data with medium/small sample size.** | ***Medium*:**<br>**Provides answers relatable to review objectives.** | ***Medium*:**<br>**A must be accredited no less than a 'medium' in 2 domains to achieve an overall WoE of Medium.** |
| | ***Low*:**<br>**1.Intervention not described.**<br>**2.Unsatisfactory methods sections for data collection and analysis with major omissions evident.**<br>**3.Unsatisfactory reporting of data and/or interpretations not warranted from findings.** | ***Low*: Study design unclear or inappropriate choice of tools used.** | ***Low*: Unable to provide answers relatable to the review objectives.** | ***Low*: A must be accredited a 'low' in 2 domains to be excluded.** |

For peer review only



For peer review only - http://bmjopen.bmj.com/site/about/guidelines.xhtml

## References

1. Institute of Medicine (US) Committee on Quality of Health Care in America. Crossing the Quality Chasm: A new healthcare system for the 21st century. Washington: National Academies Press (US); 2001.

2. Scottish Government. The Healthcare Quality Strategy for NHS Scotland. Edinburgh. Scottish Government; 2010.

3. Health Foundation. Overcoming challenges to improving quality. Lessons from the Health Foundation's improvement programme evaluations and relevant literature. London: Health Foundation; 2012.

4. Nursing and Midwifery Council. Standards for pre-registration nursing programmes. NMC. London; 2019.

5. Academy of Medical Royal Colleges. Quality improvement – training for better outcomes. Academy of Medical Royal Colleges. London; 2016.

6. QSEN Institute: Frances Payne Bolton School of Nursing. Quality and Safety Education for Nurses, 2010. Available at: 2010 (qsen.org)



For peer review only - http://bmjopen.bmj.com/site/about/guidelines.xhtml

7. Smith, F., Alexandersson, P., Bergman, B., Vaughn, L., Hellstrom, A. Fourteen years of quality improvement education in healthcare: a utilisation-focused evaluation using concept mapping. BMJ Open Quality, 2019. Available: https://doi.org: 10.1136/bmjoq-2019-000795.

8. Lillo-Crespo M, Sierras-Davó, M. C., MacRae, R., & Rooney, K. Developing a framework for evaluating the impact of Healthcare Improvement Science Education across Europe: a qualitative study. Journal of Educational Evaluation for Health Professions, 2017; 14(28). Available: https://doi.org/10.3352/jeehp.2017.14.28

9. Flynn, R., Scott, S.D., Rotter, T., Hartfield, D. The Potential for nurses to contribute to and lead improvement science in health care. Journal of Advanced Nursing, 2017; 73, p.97–107. Available: https://doi: 10.1111/jan.13164

10. Robb, G., Wells, S., Stolarek, I. and Bohm, G. The state of quality improvement and patient safety teaching in health professional education in New Zealand. New Zealand Medical Association, 2017; 130 (1464), p.13-24

11. Mak, V., Brand, G. and Morphet. J. Partnering with healthcare organisations to teach pre-registration health professions students about quality improvement: A systematic review. Journal of Clinical Nursing, 2022; p1-14.

12. Armstrong, L., Shepherd, A. & Harris, F.M. An evaluation of approaches used to teach quality improvement to preregistration healthcare professionals: an integrative



For peer review only - http://bmjopen.bmj.com/site/about/guidelines.xhtml

review. International Journal of Nursing Studies, 2017; 73, p.70-84. https://doi.org/10.1016/j.ijnurstu.2017.05.005

13. Jones, A.C., Shipman, S.A., Ogrinc, G. Key characteristics of successful quality improvement curricula in physician education: a realist review. BMJ Quality Safety, 2014; 0, p.1-12.

14. Health Foundation. Complex Adaptive Systems. London: The Health Foundation; 2010.

15. Baillie, L., Bromley, B., Walker, M., Jones, R., Mhlanga, F. Implementing service improvement projects within pre-registration nursing education: A multi-method case study evaluation. Nurse Education in Practice, 2014, 14, p. 62-68.

16. Christiansen, A., Robson, L., Griffith-Evans, C., 2010. Creating an improvement culture for enhanced patient safety: service improvement learning in pre-registration education. Journal of Nursing Management. 18, p.782-788.

17. James et al. 2014 James, B., Beattie, M., Shepherd, A., Armstrong, L., Wilkinson, J., 2016. Time, fear and transformation: Student nurses' experiences of doing a practicum (quality improvement project) in practice. Nurse Education in Practice. 19, p.70-78.



For peer review only - http://bmjopen.bmj.com/site/about/guidelines.xhtml

18. Starr, S.R., Kautz, J.M., Sorita, A., Thompson, K.M., Reed, D.A., Porter, B.L., Mapes, D.L., Roberts, C.C., Kuo, D., Bora, P.R., Elraiyah, T.A., Murad, M.H., Ting, H.H. Quality Improvement Education for Health Professionals: A systematic review. American Journal of Medical Quality, 2016. 31(3), p.209-216.

19. Beaver, P. The challenges of safety improvement in New Zealand public hospitals, Kotuitui. New Zealand Journal of Social Sciences Online, 2019; 14(1), p112.125. Available at: https://doi.org/10.1080/1177083X.2018.1561476

20. Beal, J. Academic-Service Partnerships in Nursing: An Integrative Review, Nursing Research and Practice, 2012; p.1-9. Available at: https://doi:10.1155/2012/501564

21. Macphee, M. Developing a practice-academic partnership logic model. Nursing Outlook, 2009; 57, p.143-147.

22. Brown, D., White, J., Leibrandt, L. Collaborative partnerships for nursing faculties and health service providers: what can nursing learn from business literature? Journal of Nursing Management, 2006; 14, p.170–179

23. Nguyen, V.N.B, Lawerence, K., McGillion, A. The effectiveness of partnership models in clinical nursing education – A scoping review. Nurse Education Today, 2020; 90, p.1-8



For peer review only - http://bmjopen.bmj.com/site/about/guidelines.xhtml

24. Kaplan, C.H., Brady, P.W., Dritz, M.C., Hooper, D.K., Linam, M.W., Froehle, C.M. Margolis, P.M. The influence of context on quality improvement success in 20 healthcare: A systematic Review of the Literature. The Milbank Quarterly, 2010; 88 (4), p.500-599.

25. Health Foundation. Skilled for Improvement, Learning Communities and the Skills Needed to Improve Care and Evaluate Service Development. London: Health Foundation; 2014.

26. Pawson, R., Greenhalgh, T., Harvey, G. et al. Realist Synthesis: An Introduction. ESRC Research Methods Programme, University of Manchester; 2004.

27. Skivington, K., Matthews, L., Simpson, S.A., Craig, P., Baird, J., Blazeby, J.M., Boyd, K.A., Craig, N., French, D.P., McIntosh, E., Petticrew, M., Rycroft-Malone, J., White, M., Moore, L. A new framework for developing and evaluating complex interventions: update of Medical Research Council guidance. BMJ, 2021; 374. Available at: https://doi.org/10.1136/bmj.n2061

28. Windish, D.M., Reed, D.A., Boonyasai, R.T., Chakraborti, C., Bass, E.B. Methodological rigor of quality improvement curricula for physician trainees: A systematic review and recommendations for change. Academic Medicine, 2009; 84 (12), p.1677-1692. Available at: https://doi.org/10.1097/ACM.0b013e3181bfa080



For peer review only - http://bmjopen.bmj.com/site/about/guidelines.xhtml

29. Denzin, N.K. and Lincoln, Y.S. The sage handbook of Qualitative Research. London: Sage Publications Ltd; 2005

30. Creswell, J. W. Research Design: Qualitative, Quantitative and Mixed Methods Approaches. CA: Thousand Oaks Sage; 2014

31. Bates, R. A Critical analysis of evaluation practice: the Kirkpatrick model and the principle of beneficence. Evaluation and Program Planning, 2014; 27, p.341-347

32. Armstrong, L., Shepherd, A. and Harris, F. Putting QI education into context for pre-registration nurse education: an ethnographic multiple-case study. International Forum on Quality and Safety in Healthcare 2019. Tuesday 26-29th March, The Scottish Exhibition Conference Centre, Glasgow.

33. Coles, E., Anderson, J., Maxwell, M., Harris, F.M., Gray, N.M., Milner, G., MacGillivray, S. The influence of contextual factors on healthcare quality improvement initiatives: a realist review. Systematic Reviews. 2020, 26, 9(1)

34. Kaplan, H.C., Provost, L.P., Froehle, C.M., Margolis, P.A. The model for understanding success in quality (MUSIQ): building a theory of context in healthcare quality improvement. BMJ Quality and Safety, 2012; 21, p.13–20.

For peer review only



For peer review only - http://bmjopen.bmj.com/site/about/guidelines.xhtml

35. Bate, P. Context is everything. In: Perspectives on context. London: The Health Foundation; 2014. p. 1–29.

36. Greenhalgh, J. and Manzano, A. Understanding ‘context’ in realist evaluation and synthesis. International Journal of Social Research Methodology, 2021. Available at: https://doi.org/10.1080/13645579.2021.1918484

37. Tess, A., Vidyarthi, A., Yang, J., Myers, J.S. Bridging the Gap: A framework and strategies for integrating the quality and safety mission of teaching hospitals and graduate medical education. Academic Medicine, 2015; 90 (9), p. 1251-1257.

38. Moher, D., Liberati, A., Tetzlaff, J., Altman, D.G., Prisma Group. Preferred reporting items for systematic reviews and meta-analyses: the PRISMA statement. Open Medicine, 2009; 3(2), p123-130

39. Wong, G., Greenhalgh, T., Westthorp, G., Buckingham, J., Pawson, R. RAMESES publication standards: realist syntheses. BMC Medicine, 2013; p.11-21. Available at: https://doi.org/10.1186/1741-7015-11-21

40. Abrams, R., Park, S., Wong, G. Rastogi, J, Boylan, A.M., Tierney, S., Petrova, M., Dawson, S., Roberts, N. Lost in reviews: Looking for the involvement of stakeholders, patients, public and other non-researcher contributors in realist reviews, 2020; 12(2), p. 239-247.



For peer review only

For peer review only - http://bmjopen.bmj.com/site/about/guidelines.xhtml

41. Duddy, C., Roberts, N. Identifying evidence for five realist reviews in primary health care: A comparison of search methods. Res Syn Meth. 2022;13(2):190-203. doi:10.1002/jrsm.1523

42. Pawson, R., Greenhalgh, T., Harvey, G., Walshe, K. Realist review—a new method of systematic review designed for complex policy interventions. Journal of Health Services Research and Policy, 2005; 10 p.21–34.

43. Ogrinc, G., Armstrong, G., Dolansky, M.A., Singh, M.T., Davies, L. SQUIRE-EDU (Standards for Quality Improvement Reporting Excellence in Education): Publication Guidelines for Educational Improvement. Academic Medicine, 2019; 94(10), p.1461-1470. Available at: doi: 10.1097/ACM.0000000000002750.

44. Gough, D. Weight of Evidence: A Framework for the appraisal of the quality and relevance of evidence, Research Papers in Education, 2007, pp. 213-228.

For peer review only

Figure 1 Tess et al.'s (2015) framework for integrating QI education

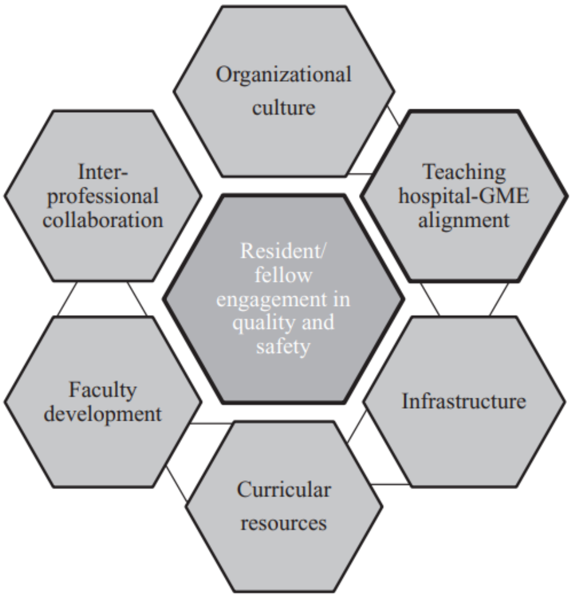


**Figure 1** Overview of a framework for integrating the quality and safety mission of a teaching hospital and graduate medical education (GME). The framework's six key elements are organizational culture, teaching hospital–GME alignment, infrastructure, curricular resources, faculty development, and interprofessional collaboration.

# PRISMA-P 2015 Checklist

**This checklist has been adapted for use with systematic review protocol submissions to BioMed Central journals from Table 3 in Moher D et al:** Preferred reporting items for systematic review and meta-analysis protocols (PRISMA-P) 2015 statement. *Systematic Reviews* 2015 **4**:1

An Editorial from the Editors-in-Chief of *Systematic Reviews* details why this checklist was adapted - **Moher D, Stewart L & Shekelle P**: Implementing PRISMA-P: recommendations for prospective authors. *Systematic Reviews* 2016 **5**:15

| Section/topic | # | Checklist item | Information reported | | Line number(s) |
|---|---|---|---|---|---|
| | | | Yes | No | |
| **ADMINISTRATIVE INFORMATION** | | | | | |
| **Title** | | | | | |
| Identification | 1a | Identify the report as a protocol of a systematic review | | Realist Review | Page 1<br>Line 3 |
| Update | 1b | If the protocol is for an update of a previous systematic review, identify as such | | No | |
| **Registration** | 2 | If registered, provide the name of the registry (e.g., PROSPERO) and registration number in the Abstract | Yes | | Page 3<br>Lines 4-5 |
| **Authors** | | | | | |
| Contact | 3a | Provide name, institutional affiliation, and e-mail address of all protocol authors; provide physical mailing address of corresponding author | Yes | | Page 1<br>Lines 1-28 |
| Contributions | 3b | Describe contributions of protocol authors and identify the guarantor of the review | Yes | | Page 17<br>Lines ?? |
| **Amendments** | 4 | If the protocol represents an amendment of a previously completed or published protocol, identify as such and list changes; otherwise, state plan for documenting important protocol amendments | | No | |
| **Support** | | | | | |
| Sources | 5a | Indicate sources of financial or other support for the review | Yes | | Page 17<br>Lines ?? |

BioMed Central
The Open Access Publisher

| Section/topic | # | Checklist item | Information reported Yes | Information reported No | Line number(s) |
|---|---|---|---|---|---|
| Sponsor | 5b | Provide name for the review funder and/or sponsor | | | Page 17<br>Lines ?? |
| Role of sponsor/funder | 5c | Describe roles of funder(s), sponsor(s), and/or institution(s), if any, in developing the protocol | | No funders | |
| **INTRODUCTION** | | | | | |
| **Rationale** | 6 | Describe the rationale for the review in the context of what is already known | Yes | | Page1<br>Lines 31-34<br>Page2<br>Lines 1-9 |
| **Objectives** | 7 | Provide an explicit statement of the question(s) the review will address with reference to participants, interventions, comparators, and outcomes (PICO) | | This is not appropriate for Realist Review methodology | |
| **METHODS** | | | | | |
| **Eligibility criteria** | 8 | Specify the study characteristics (e.g., PICO, study design, setting, time frame) and report characteristics (e.g., years considered, language, publication status) to be used as criteria for eligibility for the review | | This is not appropriate for Realist Review methodology | |
| **Information sources** | 9 | Describe all intended information sources (e.g., electronic databases, contact with study authors, trial registers, or other grey literature sources) with planned dates of coverage | Yes | | Page 12<br>Line 9-20 |
| **Search strategy** | 10 | Present draft of search strategy to be used for at least one electronic database, including planned limits, such that it could be repeated | Yes | | Page 19<br>Lines 1-37 |
| ***STUDY RECORDS*** | | | | | |

For peer review only

BioMed Central
The Open Access Publisher

| Section/topic | # | Checklist item | Information reported Yes | No | Line number(s) |
|---|---|---|---|---|---|
| Data management | 11a | Describe the mechanism(s) that will be used to manage records and data throughout the review | Yes | | Page 13<br>Lines 12-26 |
| Selection process | 11b | State the process that will be used for selecting studies (e.g., two independent reviewers) through each phase of the review (i.e., screening, eligibility, and inclusion in meta-analysis) | Yes | | Page 13-14<br>Lines 20-28<br>Lines 1-4 |
| Data collection process | 11c | Describe planned method of extracting data from reports (e.g., piloting forms, done independently, in duplicate), any processes for obtaining and confirming data from investigators | Yes | | Page 14<br>Lines 5-16 |
| **Data items** | 12 | List and define all variables for which data will be sought (e.g., PICO items, funding sources), any pre-planned data assumptions and simplifications | | This is not appropriate for Realist Review methodology | |
| **Outcomes and prioritization** | 13 | List and define all outcomes for which data will be sought, including prioritization of main and additional outcomes, with rationale | Yes | | Page 15<br>Lines 2-24 |
| **Risk of bias in individual studies** | 14 | Describe anticipated methods for assessing risk of bias of individual studies, including whether this will be done at the outcome or study level, or both; state how this information will be used in data synthesis | Yes | | Pages 14-15<br>Lines 17-28 |
| ***DATA*** | | | | | |
| **Synthesis** | 15a | Describe criteria under which study data will be quantitatively synthesized | | This is not appropriate for Realist Review methodology | |
| | 15b | If data are appropriate for quantitative synthesis, describe planned summary measures, methods of handling data, and methods of combining data from studies, including any planned exploration of consistency (e.g., $I^2$, Kendall's tau) | Yes | | Page 15<br>Lines 2-24 |
| | 15c | Describe any proposed additional analyses (e.g., sensitivity or subgroup analyses, meta- | | | n/a |

BioMed Central
The Open Access Publisher

| Section/topic | # | Checklist item | Information reported Yes | No | Line number(s) |
|---|---|---|---|---|---|
| | | regression) | | | |
| | 15d | If quantitative synthesis is not appropriate, describe the type of summary planned | | | Page 15<br>Lines 21-24 |
| **Meta-bias(es)** | 16 | Specify any planned assessment of meta-bias(es) (e.g., publication bias across studies, selective reporting within studies) | | | n/a |
| **Confidence in cumulative evidence** | 17 | Describe how the strength of the body of evidence will be assessed (e.g., GRADE) | Yes | | Page 15<br>Lines 17-28 |

For peer review only



BioMed Central
The Open Access Publisher
